# Peer Review of “Impact of the COVID-19 Pandemic on Latino Families With Alzheimer Disease and Related Dementias: Qualitative Interviews With Family Caregivers and Primary Care Providers”

**DOI:** 10.2196/56443

**Published:** 2024-03-08

**Authors:** Anna Marin

**Affiliations:** 1 Icahn School of Medicine at Mount Sinai, New York, NY, United States

**Keywords:** Latino, dementia, caregiving, COVID-19, Alzheimer’s disease, health disparity, qualitative research, transcript analysis, health inequality, minority population, epidemiology, peer support, social services, health care, Alzheimer’s, minority, qualitative, interview, caregiver, primary care, impact, resilience, disparity, outcome, Alzheimer disease, Alzheimer

*This is the peer-review report for “Impact of the COVID-19 Pandemic on Latino Families With Alzheimer Disease and Related Dementias: Qualitative Interviews With Family Caregivers and Primary Care Providers.”*

## Round 1 Review

### General Comments

This paper [1] provides an in-depth qualitative assessment of the impact and resilience factors related to the COVID-19 pandemic among Latino families with Alzheimer disease and related dementias (ADRD). The authors interviewed 21 family caregivers and 23 primary care providers (PCPs) across the United States and identified 2 primary themes that characterized the experiences of the participants, involving both the impact of the pandemic and the strategies adopted to cope with the detrimental impact of the pandemic. The topic covered is of significant importance and provides important background to better understand the impact of the COVID-19 pandemic on Latino families with ADRD, with the aim of improving the quality of care and equity among the Latino community. Overall, I think that the authors should reorganize the results to better align with the aim of the study. In my specific comments I have included specific suggestions on how to make the results more organized and succinct. To strengthen the generalizability and interpretation of the findings, the authors should also include a descriptive quantitative analysis of the interviews’ analysis, where the reader can examine the prevalence of each theme and subtheme among the 21 family members and 23 PCPs.

### Specific Comments

#### Major Comments

1. The results section of the abstract is not very clear. What are the overall findings? How have the 2 themes been identified?

2. Introduction: Please outline the qualitative variables selected to investigate the aim of the study.

3. Methods:

- Sample and assessment: Given the qualitative nature of the study, it would be helpful if the authors included an example of a question from the interview script and also attached as an appendix the template of questions they used to direct the conversation.
- Data analysis: This section is not very clear, and it would help if the authors could describe the process of coding in a step-by-step manner and also make the text more succinct.

4. Results: The authors provide a very detailed qualitative analysis; however, more quantitative information should be provided to show the prevalence of each theme and subtheme for all the caregivers and PCPs (eg, how many PCPs reported food access and malnutrition during the COVID-19 pandemic?)

#### Minor Comments

5. In the abstract, results section, please remove the capital letters for the 2 themes and consider writing them as “Qualitative analysis of transcripts revealed two themes: (1) the impact of a global pandemic (eg, accelerated cognitive and physical decline, or caregivers choosing between risking finances and the family’s infection given the work situation) and (2) developing resilience to the effects of the pandemic (eg, caregivers seeking vaccination sites, moving in with the care recipient and adopting telehealth.”

6. Introduction: This sentence should be revised for clarity: “As of January of 2022, Latinos represent 8% of the US 65 and older population, but 13% of COVID-19 cases in the same age group [9].” It is not clear if the 13% of cases is the percentage of COVID-19 cases in Latino individuals aged 65 years and older.

7. Methods:

- This sentence should be incorporated in the introduction or removed: “The goal of this study was to gain an in-depth understanding of the impact of the COVID-19 pandemic on Latino families with ADRD.”
- Who transcribed the interviews?

8. Results:

- To improve clarity, the authors should label the descriptions of the different themes as themes (1, 2, etc) and subthemes (1.1, 1.2, 1.3, etc; 2.1, 2.2, 2.3, etc). Please add this labeling both in Table 2 and in the text below to help the reader better orient into each of the themes.

9. Page 6 last sentence: Please remove “make” from “To make bring rigor and validity.”

10. Page 14, last paragraph: The authors should edit “work” with “works.”

## Round 2 Review

### General Comments

This paper provides an in-depth qualitative assessment of the impact and resilience factors related to the COVID-19 pandemic among Latino families with ADRD. The authors interviewed 21 family caregivers and 23 PCPs across the United States. They identified 2 primary themes that characterized the participants’ experiences, involving both the impact of the pandemic and the strategies adopted to cope with the detrimental impact of the pandemic. The topic covered is of significant importance and provides important background to better understand the impact of the COVID-19 pandemic on Latino families with ADRD and to improve the quality of care and equity among the Latino community. The authors did a great job improving the clarity of the methods and the results. I have included other minor revisions to further improve the clarity of the text.

### Specific Comments

#### Minor Comments

1. Results:
  - Paragraph “theme 8” (line 4): Remove “and” before “healthcare.”
  - Paragraph 8.3 (line 3): Replace “requested” with “requesting.”
2. Discussion: Please clarify the second paragraph of the “implication and future directions” section. In the first sentence, “Our findings can inform future studies. For example, participants reported pandemic-related physical and cognitive deterioration and the importance of family support.” Can you clarify what type of findings can inform future research? Could you say something on the line of “Our findings regarding the physical and cognitive deterioration caused by the pandemic and the importance of family support may help inform future studies on…” In the same paragraph, there is a very minor typo; please replace “health” with “healthy.”

## Abbreviations

ADRD: Alzheimer disease and related dementias
PCP: primary care provider
